# Duration of postoperative hyperlactatemia has predictive value in recurrent fistula after major definitive surgery for intestinal fistula

**DOI:** 10.1186/s12893-022-01465-7

**Published:** 2022-01-15

**Authors:** Ming Huang, Weiliang Tian, Shikun Luo, Xi Xu, Zheng Yao, Risheng Zhao, Qian Huang

**Affiliations:** 1 Department of General Surgery, Jiangning Hospital, Hushan Road No. 169, Nanjing, Jiangsu China; 2 Department of General Surgery, Jinling Hospital, Nanjing, Jiangsu China

**Keywords:** Recurrent fistula, Hyperlactatemia, Intestinal fistula, Outcomes

## Abstract

**Purpose:**

The present study aimed to identify the predictive value of duration of postoperative hyperlactatemia in screening patients at high risk of recurrent fistula after major definitive surgery (DS) for intestinal fistula.

**Methods:**

If the initial postoperative lactate (IPL) > 2 mmol/L, DS was defined as major definitive surgery. The 315 enrolled patients with major DS were divided into group A (2 mmol/L < IPL ≤ 4 mmol/L), group B (mmol/L < IPL ≤ 6 mmol/L), and group C (IPL > 6 mmol/L). The characteristics of patients were collected, and the duration of postoperative hyperlactatemia was analyzed. According to the occurrence of recurrent fistula (RF), patients were further divided into RF group A, and Non-RF group A; RF group B, and Non-RF group B; and RF group C, and Non-RF group C.

**Results:**

The duration of postoperative hyperlactatemia was comparable between the RF group A and the Non-RF group A [12 (IQR: 12–24) vs 24 (IQR: 12–24), p = 0.387]. However, the duration of hyperlactatemia was associated with RF in group B (adjusted OR = 1.061; 95% CI: 1.029–1.094; p < 0.001) and group C (adjusted OR = 1.059; 95% CI: 1.012–1.129; p = 0.017). In group B, the cutoff point of duration of 42 h had the optimal predictive value (area under ROC = 0.791, sensitivity = 0.717, specificity = 0.794, p < 0.001). In group C, the cutoff point of duration of 54 h had the optimal predictive value (area under ROC = 0.781, sensitivity = 0.730, specificity = 0.804, p < 0.001).

**Conclusion:**

The duration of postoperative hyperlactatemia has a value in predicting RF in patients with an IPL of more than 4 mmol/L after major definitive surgery for intestinal fistula.

## Introduction

Intestinal fistula is a potentially life-threatening pathological condition [1], which results from inflammatory bowel disease (IBD)^2^, malignant tumor [3], intestinal radiation injury [4], and iatrogenic causes [5]. With good infection control and nutritional support, spontaneous closure might occur in a considerable number of patients [5, 6]; however, definitive surgical treatment is the main treatment method [7]. In patients who undergo a complicated surgical process, the process can be quite difficult and bumpy [7], and there could be a very high incidence of complications [7, 8]. In our previous study, the rate of recurrent fistula (RF) was as high as 30% after complicated definitive surgery [7]. As a result, exploration of the possible factors of RF is an important topic of surgical treatment. Lactate is widely used as a biomarker for screening, diagnosis, risk stratification, and monitoring in critically ill patients [9]. In addition, it was found that postoperative hyperlactatemia had an association with complications after abdominal surgery [10]. Factors associated with elevated epinephrine levels, such as not only inadequate intraoperative rehydration, but also an aggravating inflammatory reaction following intra-operative extensive stress, would lead to postoperative hyperlactatemia after major surgery [11]. In complicated definitive surgery for intestinal fistula, massive blood loss, long operation time, and serious intraoperative injury [8, 12] lead to relative long-term hyperlactatemia following severe postoperative stress and a relative long-term hormone secretion disorder. Postoperative hyperlactatemia is associated with microcirculatory damage, leading to a reduction in the ability of tissue to extract oxygen [13], which might have an influence on healing of the anastomotic site after definitive surgery for intestinal fistula and lead to RF. In the present study, we aimed to identify the value of duration of postoperative hyperlactatemia in screening patients at high risk of intestinal fistula.

## Methods and materials

This is a retrospective study, and it was approved by the Institutional Review Board. All methods were performed in accordance with the relevant guidelines and regulations. Informed consent was obtained from all individuals.

### Study design

From January 2015 to December 2019, the patients who underwent major definitive surgery for intestinal fistula were enrolled. If the initial postoperative lactate level > 2 mmol/L, definitive surgery was defined as major definitive surgery. The exclusion criteria included: (1) patients younger than 18 years; (2) patients with fistulas in different anatomical positions; (3) patients with IBD; and (4) patients without complete data. The enrolled patients were divided into group A (initial postoperative lactate level > 2 mmol/L and ≤ 4 mmol/L), group B (initial postoperative lactate level > 4 mmol/L and ≤ 6 mmol/L), and group C (initial postoperative lactate level > 6 mmol/L). According to the occurrence of RF, patients were further divided into RF group A, and Non-RF group A; RF group B, and Non-RF group B; and RF group C, and Non-RF group C. The characteristics of patients were collected and analyzed. The value of duration of hyperlactatemia in predicting RF after complicated definitive surgery for intestinal fistula was evaluated.

### Definitive surgery

After admission, all patients were kept fasting and not allowed to drink water. Before planning the definitive surgery, the abdominal infection was gradually controlled, and enteral nutrition was gradually established via a nasointestinal tube or nasogastric tube. In brief, the criteria to plan definitive surgery in patients were as follows: (1) abdominal infection due to a controlled fistula for more than 3 months (during treatment, patients developed venous disease and cholecystitis due to long-term fasting. Although the infection was controlled for a long time in these patients, after these episodes, the infection indexes needed to attain a normal level for one month till surgery); (2) Enteral nutrition provided more than 80% energy for at least 3 months; (3) BMI ≥ 18 kg/m^2^ and normal physical strength; and (4) hemoglobin ≥ 110 g/L.

In our study, we defined definitive surgery for intestinal fistula as follows: Continuity of the digestive tract could be restored by surgery in a single session. In other words, in our study, the fistulas were resected or repaired and there was no permanent stoma or protective stoma, if it satisfied with definitive surgery. During surgery, the digestive tract was gradually dissociated. For different fistulas, the surgical process was different. Latero-lateral end anastomosis was performed for every small intestinal fistula using a linear stapler (Pride Medical Inc., Jingjiang, Taizhou, Jiangsu, China). End-to-end anastomosis was performed using a curved intraluminal stapler (Ethicon, Endo-Surgery, New Mexico, USA) in patients with ileocolic anastomotic fistula, fistula at the appendix stump, or colonic fistula. Gastric fistula was closed using a linear stapler (Pride Medical Inc., Jingjiang, Taizhou, Jiangsu, China). On the other hand, repair using a 4–0 absorbable band (VICRYL plus, Ethicon, Inc., Texas, USA) was performed at each duodenal fistula and gastrojejunal anastomotic fistula. Besides definitive surgery, all patients with enteroatmospheric fistula underwent hernia repair during surgery. Components separation technology + onlay mesh repair was used. During the procedure, a Cook Biodesign advanced tissue repair product (Cook Medical Inc., Bloomington, IN, USA) was used. A negative pressure drainage system was placed under all incisions before the operation was completed.

### Anatomical positions of fistulas

The anatomical positions of fistulas included the following: duodenum, stomach, gastrojejunal anastomosisc, jejunum/ ileum, ileocolonic anastomosis or appendiceal stump, and colon (Table 1). Ileocolonic anastomotic fistula and fistula at the appendiceal stump were considered as one entity because of the same surgical management. In our study, each enrolled patient had fistula(s) in only one anatomical position.

**Table 1:** Baseline characteristics and univariate analysis

	RF group A (n = 7)	Non-RF group A (n = 46)	p	RF group B (n = 53)	Non-RF group B (n = 126)	p	RF group B (n = 37)	Non-RF group B (n = 46)	p
Female, No. (%)	3 (42.86)	25 (54.35)	0.570	26 (49.06)	67 (53.17)	0.615	15 (40.54)	21 (45.65)	0.640
Age, year (median, IQR)	56 (44–60)	54 (37–58)	0.107	52 (39–60)	53 (36–63)	0.751	56 (37–67.5)	54 (39–65)	0.620
BMI, kg/m^2^ (mean ± SD)	20 (19–20.5)	19.5 (19–20.5)	0.847	20 (19–21)	20 (19–21)	0.576	20 (19–20)	19.5 (19–21)	0.328
Etiology, No. (%)			0.431			0.489			0.766
Trauma	4 (57.14)	14 (30.43)		15 (28.30)	32 (25.39)		18 (48.64)	19 (41.30)	
Obstruction due to previous surgery^a^	0	9 (19.56)		5 (9.43)	20 (15.87)		2 (5.41)	2 (4.35)	
Mesenteric thrombosis	0	2 (4.35)		2 (3.77)	4 (3.17)		2 (5.41)	1 (2.17)	
Tumour	3 (42.86)	16 (34.78)		11 (20.75)	45 (35.71)		6 (16.21)	12 (26.08)	
Appendicitis	0	5 (10.87)		0	0		0	0	
Pancreatitis	0	0		12 (22.64)	25 (19.84)		9 (24.32)	12 (26.09)	
Location of the fistula, No. (%)			0.412			0.113			0.102
Duodenum	3 (42.86)	14 (30.43)		22 (41.51)	40 (31.75)		18 (48.64)	17 (36.96)	
Stomach	1 (14.29)	4 (8.69)		0	4 (3.17)		1 (2.7)	0	
Gastrojejunal anastomotic	1 (14.29)	0		7 (13.20)	9 (7.14)		7 (18.92)	4 (8.69)	
Jejunum/ileum	0	12 (26.08)		9 (16.98)	40 (31.75)		4 (10.81)	16 (34.78)	
Ileocolonic anastomosis or appendiceal stump^b^	0	11 (23.91)		1 (1.89)	7 (5.55)		1 (2.7)	3 (6.52)	
Colon	2 (28.57)	5 (10.87)		14 (26.41)	26 (20.63)		6 (16.21)	6 (13.04)	
Interval from fistula occurred to admission, days, (median, IQR)	21 (14–30)	23 (14–29)	0.182	23 (20–32)	23 (20–30)	0.575	24 (20–34)	24 (22–34)	0.771
Interval from fistula occurred to definitive surgery, days, (median, IQR)	125 (105–141)	120 (102–129)	0.198	121 (109–140)	120 (105–138)	0.509	126 (114–144)	122 (110–135)	0.176
Enteroatmospheric fistula, No. (%)	2 (28.57)	3 (6.52)	0.124	22 (41.51)	43 (34.12)	0.384	22 (59.46)	19 (41.30)	0.100
Preoperative albumin, g/L, (median, IQR)	37 (35–40)	38 (36–39)	0.690	38 (36–39)	37 (36–39)	0.695	37 (36–38)	38 (36–39)	0.107
Preoperative hemoglobin, g/L, (median, IQR)	112 (110–125)	119 (111–125)	0.867	117 (110–124)	121 (115–126)	0.035	118 (114–126)	121 (114–124)	0.656
Grade of abdominal adhesions, No. (%)			0.031			0.037			0.576
III	3 (42.86)	37 (80.43)		7 (13.21)	14 (11.11)		N/A	N/A	
IV	4 (57.14)	9 (19.56)		26 (49.06)	86 (68.25)		10 (27.02)	10 (21.74)	
V	N/A	N/A		20 (37.74)	26 (23.02)		27 (72.98)	36 (78.26)	
Blood loss during definitive surgery, mL (median, IQR)	800 (700–1100)	600 (500–700)	< 0.001	1500 (1250–1600)	1250 (1000–1537)	0.002	2250 (1650–2850)	2041 (1265–2633)	< 0.001
Duration of definitive surgery, min (median, IQR)	163 (141–170)	138 (131–150)	0.003	228 (211–246)	230 (206–253)	0.534	278 (227–315)	268 (234–305)	0.985
Intraoperative red blood cell transfusion, Unit (median, IQR)	2 (2–3)	2 (2–2)	0.671	4 (4–4)	4 (4–4)	0.359	5 (4–6)	5 (4–6)	0.599
Postoperative red blood cell transfusion within 48 h after definitive surgery^b^, Unit (median, IQR)	3 (3–4)	2 (0–3)	0.001	6 (6–7)	4 (4–6)	< 0.001	10 (6–11)	9 (5–11)	< 0.001
Postoperative Albumin transfusion within 48 h after definitive surgery^c^, g (median, IQR)	60 (40–80)	40 (20–50)	0.003	100 (85–160)	120 (120–180)	0.008	200 (100–210)	180 (170–190)	< 0.001
Postoperative positive fluid balance 48 h after surgery, No. (%)	3 (42.86)	8 (17.39)	0.122	24 (19.04)	30 (23.81)	0.004	28 (75.68)	26 (56.52)	0.069
Time for lactate recovery to < 2 mmol/L after definitive surgery, hours (median, IQR)	24 (12–24)	12 (12–24)	0.387	48 (IQR 48–72)	24 (IQR: 24–48)	p < 0.001	72 (IQR 48–72)	48 (IQR:24–48)	p < 0.001
Comorbidity, No. (%)									
Hypertension	0	2 (4.35)	1.000	5 (9.43)	10 (7.93)	0.302	4 (10.81)	5 (10.87)	1.000
Diabetes mellitus	1 (14.28)	0	0.132	6 (11.32)	7 (5.56)	0.175	6 (16.22)	4 (8.69)	0.295
Chronic hepatitis	1 (14.29)	1 (2.17)	0.249	2 (3.77)	4 (3.17)	1.000	2 (5.41)	1 (2.17)	0.583

^a^Before admission, the patient received surgical treatment of intestinal obstruction following a previous abdominal surgery

^b^In order to maintain the Hemoglobin > 100 g/L within 48 h after definitive surgery

^c^In order to maintain the Albumin > 30 g/L within 48 h after definitive surgery

### Data analyses

The baseline data, including demography and characteristics of fistula, were collected. Preoperative blood routine and biochemical results were recorded within 3 days before definitive surgery. The method of determining lactate was the blood gas analysis. The initial postoperative lactate level was measured when the patient was admitted to the intensive care unit (ICU). The lactate level was measured every 12 h routinely. The postoperative blood routine and biochemistry were measured every 24 h after surgery.

All statistical analyses were performed using the SPSS 26.0 (IBM, Analytics, Armonk, NY, USA). The Mann–Whitney U test was used to compare continuous variables across groups. The Fisher’s exact test was used to compare categorical variables. Multivariate logistic regression analysis was used to compare the effects of different methods. Area under the curve of the receiver operating characteristic curve (AUCROC) analysis was used to define the optimal cutoff point for predicting RF. A P value of < 0.05 was considered to indicate statistical significance.

## Results

### Population

**Fig. 1:**
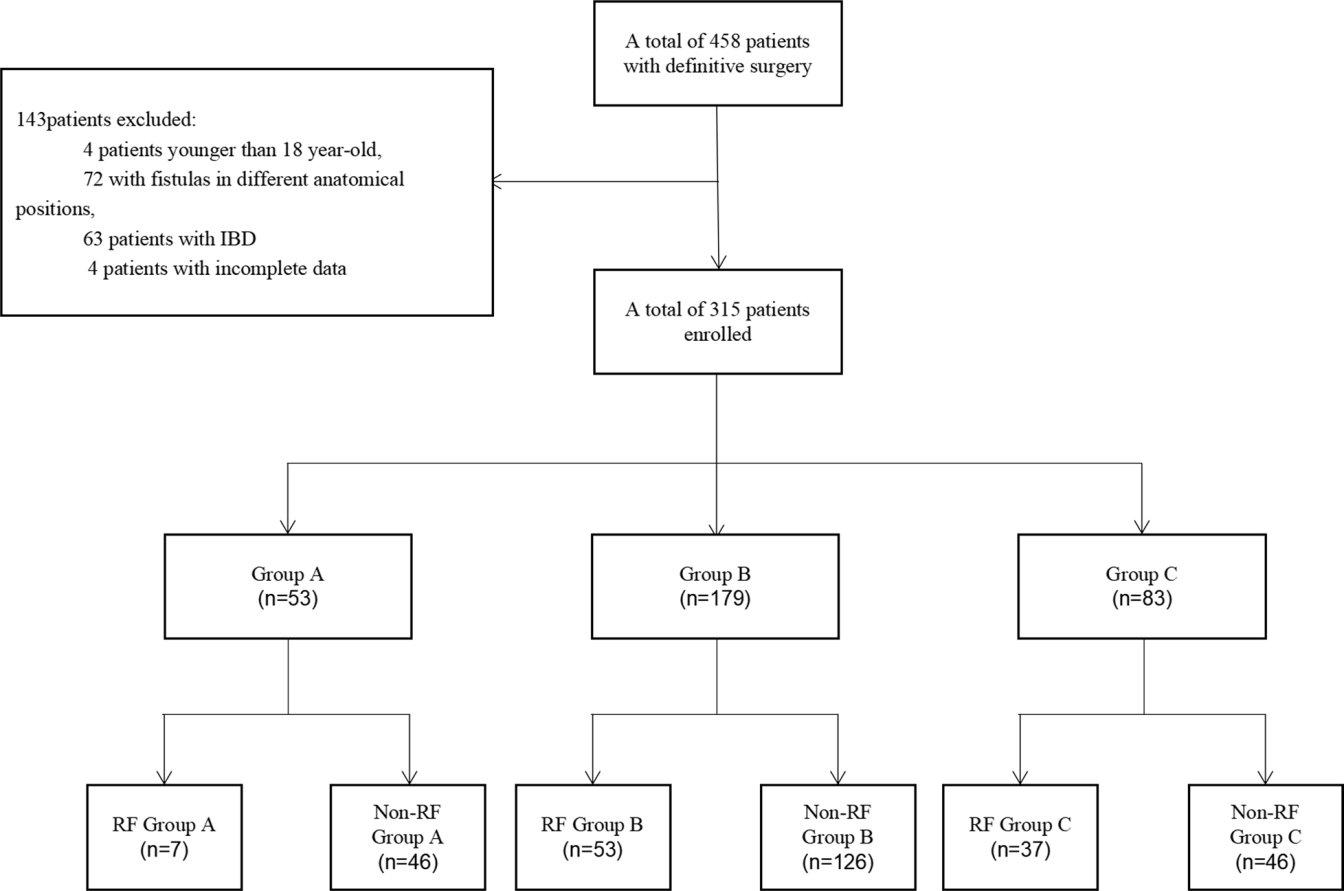
The grouping of the 315 patients

From January 2017 to December 2019, there were 458 patients received a definitive surgery for intestinal fistula. One hundred and forty-three (four patients younger than 18 year-old, 72 patients with fistula in different anatomical positions, 63 patients with IBD,and four patients with incomplete data) of the 458 patients were excluded. A total of 315 patients were enrolled (Table 1). In these 315 patients, there were a total of 150 females (47.62%) and 165 males (52.38%). The median age was 53 (IQR: 37–63) years (Fig. 1). The number of patients in groups A, B, and C was 53, 179, and 83, respectively. The incidence of RF in groups A, B, and C was 13.21% (7/53), 29.61% (53/179), and 44.58% (37/83), respectively (p < 0.001).

### Overall analysis of the duration of postoperative hyperlactatemia in 315 patients

**Fig. 2:**
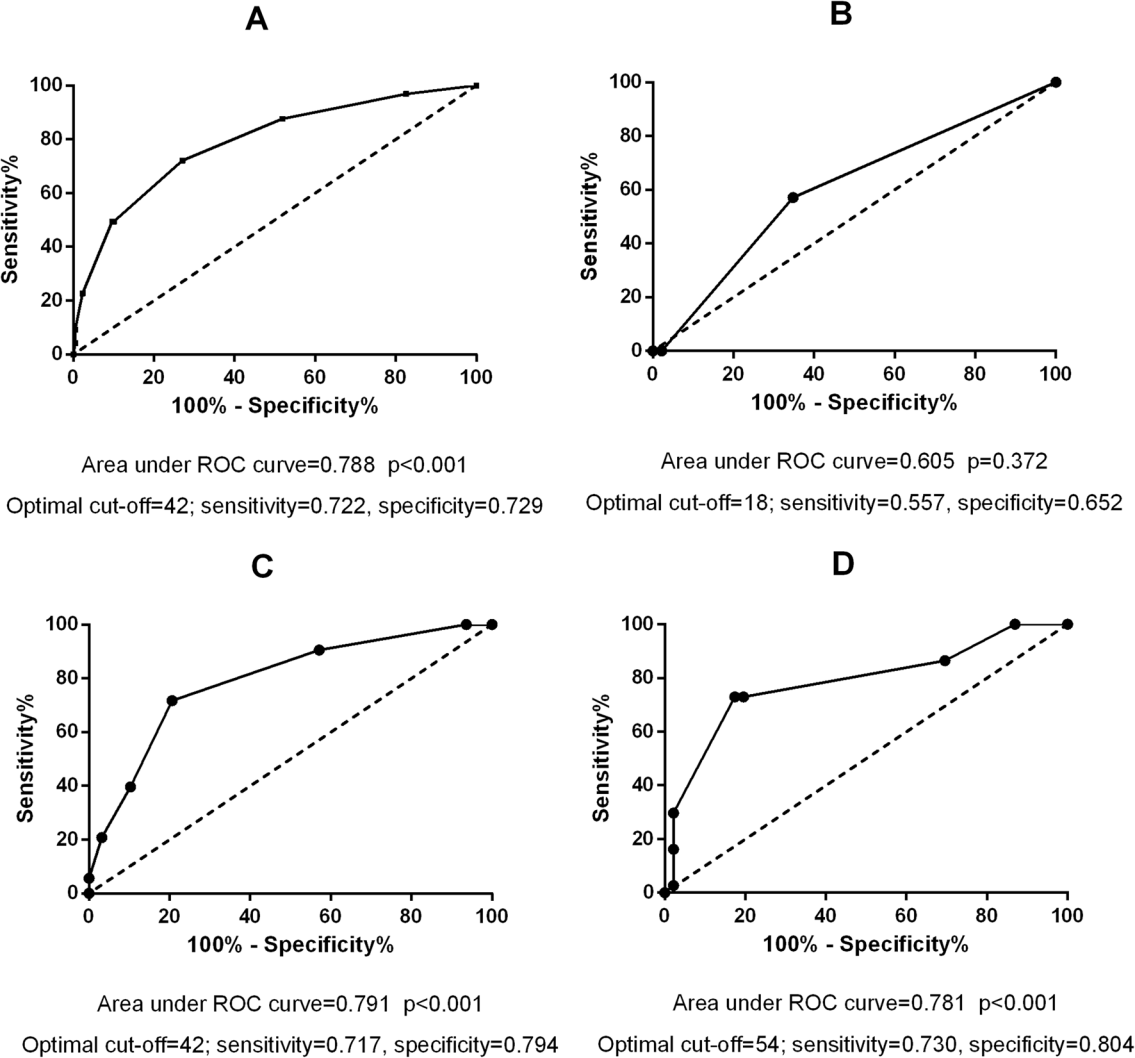
**A** the AUCROC analysis for duration of hyperlactatemia in the total 315 patients. **B** the AUCROC analysis for duration of hyperlactatemia in the Group A. **C** the AUCROC analysis for duration of hyperlactatemia in the Group B. **D** the AUCROC analysis for duration of hyperlactatemia in the Group C

**Fig. 3:**
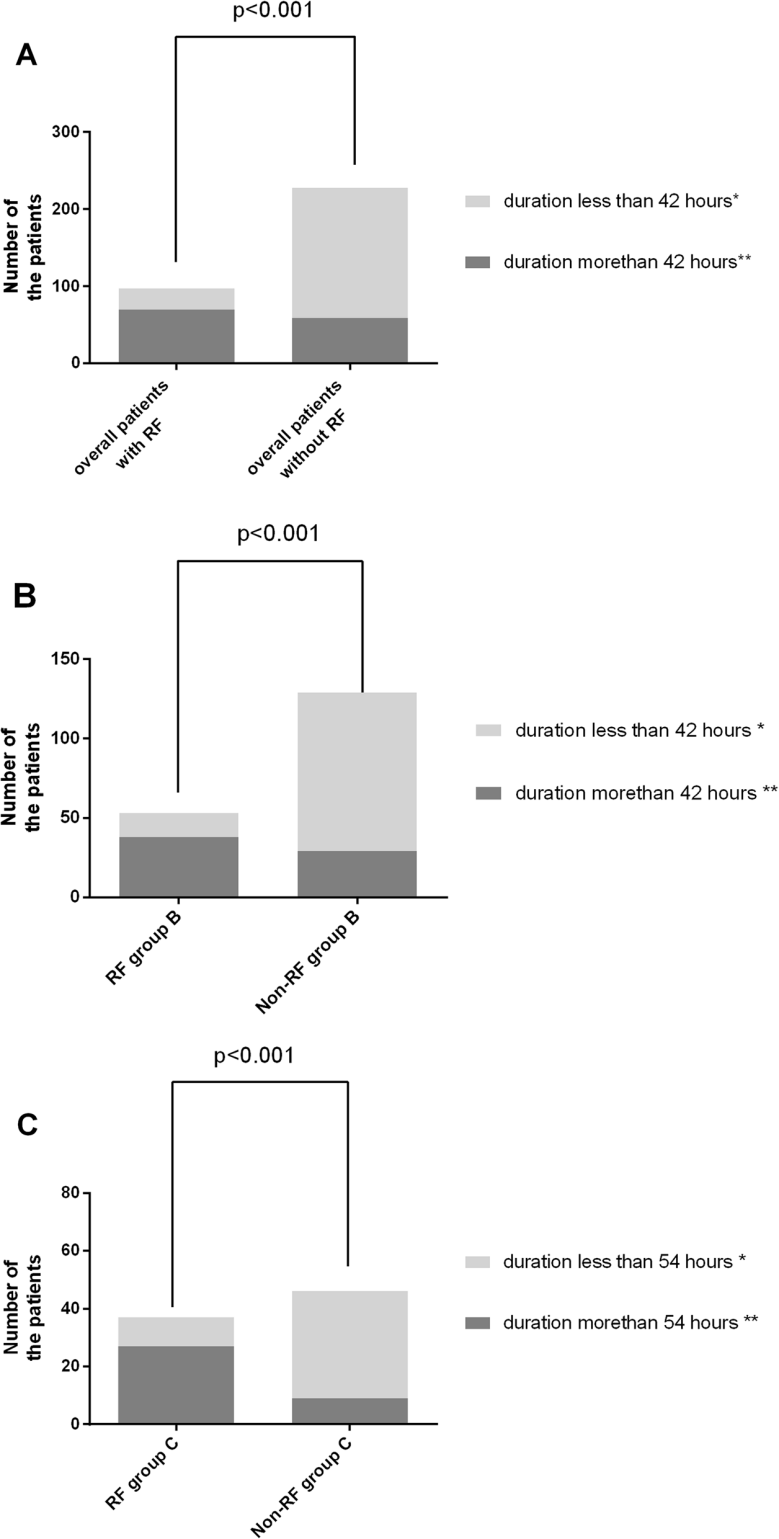
The proportion of patients who endure postoperative hyperlactatemia for more than 42 h and less than 42 h after definitive surgery between the patients with RF (n = 97) and without RF (n = 218). *number of patients with RF vs number of patients without RF = 27 (37.84%) vs 159 (72.94%); **number of patients with RF vs number of patients without RF = 70 (61.86%) vs 59(27.06%); The proportion of patients who endure post operative hyperlactatemia for more than 42 h and less than 42 h after definitive surgery between the RF group B (n = 53) and Non-RF group B (n = 126). *number of patients in RF group B vs number of patients in Non-RF group B = 15 (28.31%) vs 100 (79.37%); **number of patients in RF group B vs number of patients in Non-RF group B = 38 (71.69%) vs 26 (20.63%). **A** The proportion of patients who endure postoperative hyperlactatemia for more than 54 h and less than 54 h after definitive surgery between the RF group C (n = 37) and Non-RF group C (n = 46). *number of patients in RF group C vs number of patients in Non-RF group C = 10 (27.03%) vs 37 (80.43%); **number of patients in RF group C vs number of patients in Non-RF group C = 27 (72.97%) vs 9 (19.57%)

The overall incidence of RF in these 315 patients was 30.79% (n = 97). Initial postoperative lactate (5.16 ± 2.42 mmol/L vs 4.01 ± 2.12 mmol/L, p < 0.001)and, the duration of hyperlactatemia was significantly higher in patients with RF. Multivariate analysis in these 315 patients showed that the duration of hyperlactatemia was associated with RF (OR = 1.056; 95% CI: 1.033–1.078; p < 0.001, Table 2). AUCROC analysis demonstrated that the cutoff point of duration of 42 h had the optimal predictive value for RF (area under ROC = 0.788, sensitivity = 0.722, specificity = 0.729, p < 0.001; Fig. 2A). A total of 129 of these 315 patients had a duration of hyperlactatemia of more than 42 h. A total of 54.26% (n = 70) of these 129 patients were correctly diagnosed with RF (Fig. 3A).

**Table 2:** Multivariate inter-group logistics analysis for recurrent fistula in all of the 315 patients

	OR	95% CI	p
Location of the fistula			
Duodenum	Ref.		
Stomach	0.916	0.066–12.627	0.948
Gastrojejunal anastomotic	1.422	0.443–4.570	0.554
Jejunum/ileum	0.704	0.309–1.605	0.404
Ileocolonic anastomosis	0.619	0.119–3.223	0.569
Colon	1.677	0.695–4.046	0.250
Blood loss during definitive surgery	1.001	0.998–1.105	0.173
Duration of definitive surgery	0.994	0.985–1.004	0.256
Postoperative red blood cell transfusion within 48 h after definitive surgery^a^	1.002	1.001–1.003	0.001
Postoperative Albumin transfusion within 48 h after definitive surgery^b^	1.005	0.986–1.024	0.586
Duration of hyperlactemia after definitive surgery	1.056	1.033–1.078	< 0.001
Initial postoperative lactate	1.202	1.022–1.872	0.027
Diabetes mellitus	2.471	1.165–5.271	0.020

^a^In order to maintain the Hemoglobin > 100 g/L within 48 h after definitive surgery

^b^In order to maintain the Albumin > 30 g/L within 48 h after definitive surgery

### Intra-group analysis of the duration of hyperlactatemia among groups A

The changes in lactate levels in groups A, B, and C are shown in Table 3. The lactate level (2.88 ± 0.48 mmol/L vs 2.60 ± 0.37 mmol/L, p = 0.077) and the duration of hyperlactatemia after surgery was comparable between RF group A and Non-RF group A [12 (IQR: 12–24) vs 24 (IQR: 12–24), p = 0.387 (Table 3)]. In addition, the AUCROC analysis demonstrated that, in group A, the cutoff point of duration of 18 h did not have an optimal predictive value (area under ROC = 0.605, sensitivity = 0.557, specificity = 0.652, p = 0.372; Fig. 2B).

**Table 3:** Changes of lactate

	RF Group A	Non-RF Group A	p	RF Group B	Non-RF Group β	p	RF Group C	Non-RF Group C	p
Postoperative lactate, mmol/L, (median, IQR)	2.88 ± 0.48	2.60 ± 0.37	0.077	5.05 ± 0.62	4.82 ± 0.54	0.015	7.17 ± 0.47	6.45 ± 0.66	< 0.001
Postoperative lactate at 12th hour after definitive surgery, mmol/L, (median, IQR)	2.08 ± 0.28	1.98 ± 0.34	0.456	3.44 ± 0.52	3.16 ± 0.63	0.006	4.52 ± 1.14	3.76 ± 0.93	0.001
Postoperative lactate at 24th hour after definitive surgery, mmol/L, (median, IQR)	1.91 ± 0.18	1.64 ± 0.30	0.028	2.29 ± 0.54	2.08 ± 0.51	0.014	2.86 ± 0.47	2.41 ± 0.73	< 0.001
Postoperative lactate at 48th hour after definitive surgery, mmol/L, (median, IQR)	1.40 ± 0.25	1.26 ± 0.33	0.329	1.85 ± 0.34	1.72 ± 0.34	0.023	2.08 ± 0.46	1.81 ± 0.26	0.001
Postoperative lactate at 72th hour after definitive surgery, mmol/L, (median, IQR)	1.31 ± 0.43	1.20 ± 0.29	0.373	1.67 ± 0.46	1.49 ± 0.52	0.035	1.92 ± 0.46	1.66 ± 0.37	0.007
Postoperative lactate at 7th day after definitive surgery, mmol/L, (median, IQR)	1.10 ± 0.40	1.16 ± 0.41	0.716	1.23 ± 0.45	1.15 ± 0.45	0.302	1.21 ± 0.38	1.06 ± 0.39	0.088

### Intra-group analysis of the duration of hyperlactatemia among groups B

Initial postoperative lactate levels were higher in RF group B (5.05 ± 0.62 mmol/L vs 4.82 ± 0.54 mmol/L, p = 0.015). The duration of hyperlactatemia was longer in RF group B [48 (IQR 36–60) vs 36 (IQR: 24–36), p < 0.001] (Table 3). Multivariate analyses found that the duration of hyperlactatemia was associated with RF in group B (OR = 1.061; 95% CI: 1.029–1.094; p < 0.001) (Table 4). The AUCROC analysis demonstrated that, in group B, the cutoff point of duration of 42 h had an optimal predictive value (area under ROC = 0.791, sensitivity = 0.717, specificity = 0.794, p < 0.001. Figures 2C, 3B).

**Table 4:** Multivariate intra-group logistics analysis for recurrent fistula of the A, B and, C group

	Intra-group analysis in group A (n = 53)			Intra-group analysis in group B (n = 179)			Intra-group analysis in group C (n = 83)		
	OR	95% CI	p	OR	95% CI	p	OR	95% CI	p
Grade of abdominal adhesions, No. (%)							N/A	N/A	N/A
III	Ref.			Ref.			N/A	N/A	N/A
IV	5.871	1.159–21.887	0.032	1.672	0.998–9.901	0.673			
V	–	–	–	2.631	0.342–8.403	0.380			
Blood loss during definitive surgery	1.009	0.994–1.082	0.146	1.000	0.999–1.002	0.714	1.007	1.001–1.013	0.020
Initial postoperative lactate	N/A	N/A	N/A	1.521	0.998–1.98	0.096	1.484	0.991–1.989	0.190
Duration of definitive surgery	1.073	0.980–1.175	0.127	N/A	N/A	N/A	N/A	N/A	N/A
Postoperative red blood cell transfusion within 48 h after definitive surgery^a^	2.144	0.358–7.619	0.301	1.506	1.310–3.409	< 0.001	1.001	0.999–1.002	0.438
Postoperative Albumin transfusion within 48 h after definitive surgery^b^	1.001	0.927–1.079	0.973	1.053	1.018–1.089	< 0.001	1.068	1.028–1.108	0.001
Postoperative positive fluid balance 48 h after surgery	N/A	N/A	N/A	2.054	1.316–7.952	0.002	N/A	N/A	N/A
Duration of hyperlactemia after definitive surgery	N/A	N/A	N/A	1.061	1.029–1.094	< 0.001	1.059	1.012–1.129	0.017

^a^In order to maintain the Hemoglobin > 100 g/L within 48 h after definitive surgery

^b^In order to maintain the Albumin > 30 g/L within 48 h after definitive surgery

### Intra-group analysis of the duration of hyperlactatemia among groups C

Initial postoperative lactate levels were higher in RF group C (7.17 ± 0.47 mmol/L vs 6.45 ± 0.66 mmol/L, p < 0.001). The duration of hyperlactatemia was longer in RF group C [60 (IQR 48–72) vs 48 (IQR: 36–48), p < 0.001]. (Table 3). Multivariate analyses found that the duration of hyperlactatemia was associated with RF in group C (OR = 1.059; 95% CI: 1.012–1.129; p = 0.017) (Table 4). The AUCROC analysis demonstrated that, in group C, the cutoff point of duration of 54 h had an optimal predictive value (area under ROC = 0.781, sensitivity = 0.730, specificity = 0.804, p < 0.001. Figures 2D, 3C).

## Discussion

We conducted a retrospective subgroup study that investigated the influence of not only the initial postoperative lactate level but also the duration of postoperative hyperlactatemia on RF in patients treated with definitive surgery for intestinal fistula. It was found that, in patients with an initial postoperative lactate level more than 4 mmol/L (groups B and C), the duration of hyperlactatemia was associated with RF; however, in patients with an initial postoperative lactate level less than 4 mmol/L, the duration of hyperlactatemia had no value in predicting RF.

In previous studies, the postoperative lactate level was associated with postoperative outcomes after gastrointestinal surgery. Ben C Creagh-Brown et al. [14] investigated more than 100,000 people after gastrointestinal surgery, and they found that the postoperative peak lactate level within 24 h was independently associated with in-hospital mortality and postoperative length of stay in the hospital. Shimazaki et al. [15] found that the postoperative lactate level was a mortality marker in patients with colorectal perforation. In addition to the initial postoperative or peak lactate level, researchers started to pay attention to the association between change in the postoperative lactate level and prognosis. Veličković et al. [10] investigated the change in the postoperative lactate level at the 4th, 12th, and 24th hour after abdominal surgery, and it was found that the lactate level at the 12th hour was the most valuable to predict the prognosis. Li et al. [16] conducted a small prospective study and found that dynamic changes in blood lactate levels during the first 24 postoperative hours were significantly associated with complications after major elective abdominal surgery. In those previous studies the postoperative hyperlactemia within 24 h might a response to the severity of the surgical trauma, which might be associated the postoperative outcomes. However, in our study,all the patients had severe surgical trauma. Maybe in this cases, the recovery was more important for RF. And the duration of postoperative hyperlactemia might be a reflect on the recovery.

The process of complicated definitive surgery for intestinal fistula seems to be an abdominal surgeon's nightmare. During the process, the anatomical structure is disordered, the adhesion is severe, and there is a large amount of extensive bleeding in the operation field [7, 8]. The above factors cause severe postoperative stress in the patients; thus, leading to release of cytokines and inflammatory mediators [including tumor necrosis factor alpha (TNF-α), and several interleukins (IL-1, IL-6, and IL-8)] [17]. Along with the upregulation of proinflammatory cytokines and acute phase proteins in response to surgical stress, there is activation of the hypothalamic–pituitary–adrenal axis that leads to endocrine and metabolic disorders [18]. In addition, the increased inflammatory response following surgery leads to the suppression of mitochondrial activity and damage of mitochondria [18]. Moreover, the release of inflammatory factors can damage endothelial cells and aggravate microcirculatory disturbance [19]. These pathological processes lead to tissue hypoxia and ischemia [11]. Direct manifestation of postoperative hyperlactatemia is tissue hypoxia. Adequate oxygen supplementation is critical for nearly all wound healing processes. It prevents wounds from infection; induces angiogenesis; increases keratinocyte differentiation, migration, and re-epithelialization; enhances fibroblast proliferation and collagen synthesis; and promotes wound contraction [20, 21]. In addition, the level of reactive oxygen species (ROS) is critically dependent on the oxygen levels, which is thought to act as cellular messengers to stimulate key processes associated with wound healing [22]. As an important index, the duration of hyperlactatemia has an obvious effect on tissue healing and prediction of RF.

In addition, it is well known that the first 48 h after injury is the crucial period for neutrophils to enter the injured area through capillaries. During this period, microcirculatory disturbance will undoubtedly affect the infiltration of inflammatory cells. In the present study, when the postoperative lactate level was less than 4 mmol/L, the lactate level could recover to the normal level within postoperative 12 h in most cases, and in addition, it could recover to the normal level within postoperative 24 h in all cases. It seemed that the patients with an initial postoperative lactate level less than 4 mmol/L had too short duration of hyperlactatemia to influence the tissue healing process. As a result, the duration was not associated the RF. On the other hand, in the present study most patients with RF had a duration of hyperlactatemia of more than postoperative 42 h. The duration was long enough to influence the tissue healing process by influencing invasion of inflammatory cells. A common view is that blood loss and postoperative blood delivery majorly affect initial postoperative lactate. However, the subgroup analysis showed that the initial postoperative lactate did not have influence on RF, while the duration of postoperative hyperlactatemia had predictive value to a considerable number of patients. Furthermore, in the present study, in addition to the extensive bleeding following tissue dissection, the vascular injury was also the cause of bleeding. Compared with bleeding following vascular injury, the extensive bleeding have a greater negative influence on postoperative microcirculation and inflammation. It further affect the incidence of postoperative RF. As the result, avoiding the cause of bleeding, blood loss and postoperative blood delivery alone does not predict RF.

Of course, there seems to be another explanation for the influence of duration of hyperlactatemia on RF. For instance, postoperative surgical stress could lead to metabolic disorders. Hyperlactatemia might be one of the manifestations of metabolic disorders, and other manifestations include hyperdecomposition state and hyperglycemia, which might influence RF. However, in our study, postoperative parenteral nutrition and blood glucose control were strictly conducted. As a result, these influences should be investigated in the further study.

There were limitations to our study. First, as this study was a retrospective study, and the sample size of our study was small. Selection bias existed. Second, causes of fistula were diverse in our study. The etiological diversity may be part of the less power of the study. Third, due to the insufficient data, we did not evaluate the influence of evolution of the fistulas on result. It might be evaluated in future studies. Fourth our data of the postoperative lactate level were collected every 12 h after definitive surgery. The interval between two measuring points seemed a little long, and this might lead to bias. More accurate postoperative lactate monitoring can be implemented in future prospective studies. In addition, our wards have their own ICU and were managed by surgeons instead of doctors in critical care medicine. Our fluid resuscitation strategy seemed to be conservative (vasoactive drugs are priority). This was likely to have a negative effect on lactic acid recovery.

## Conclusion

The duration of postoperative hyperlactatemia has a value in predicting RF in patients with an initial postoperative lactate level more than 4 mmol/L after major definitive surgery for intestinal fistula.

## Data Availability

The datasets used and/or analysed during the current study available from the corresponding author on reasonable request.

## Abbreviations

DS: Definitive surgery
IBD: Bowel disease
ICU: Intensive care unit
IL: Interleukins
IPL: Initial postoperative lactate
RCO: Receiver operating characteristic
RF: Recurrent fistula
ROS: Reactive oxygen species
TNF-α: Tumor necrosis factor alpha

## References

[1] Hatchimonji JS, Passman J, Kaufman EJ (2020). Enterocutaneous fistula after emergency general surgery: mortality, readmission, and financial burden. J Trauma Acute Care Surg.

[2] Torres J, Mehandru S, Colombel JF, Peyrin-Biroulet L (2017). Crohn’s disease. Lancet.

[3] Berry SM, Fischer JE (1996). Classification and pathophysiology of enterocutaneous fistulas. Surg Clin North Am.

[4] Zelga P, Tchórzewski M, Zelga M, Sobotkowski J, Dziki A (2017). Radiation-induced rectovaginal fistulas in locally advanced gynaecological malignancies-new patients, old problem?. Langenbecks Arch Surg.

[5] Quinn M, Falconer S, McKee RF (2017). Management of enterocutaneous fistula: outcomes in 276 patients. World J Surg.

[6] Yang F, Liu D, Xu X (2020). A double-lumen irrigation-suction tube placed during operation could reduce the risk of grade C anastomotic leakage resulting from selective sigmoid colon cancer radical resection. Langenbecks Arch Surg.

[7] Tian W, Xu X, Yao Z (2021). Early enteral nutrition could reduce risk of recurrent leakage after definitive resection of anastomotic leakage after colorectal cancer surgery. World J Surg.

[8] Tian W, Yan M, Xu X, Yao Z, Zhao R (2021). Risk factors and outcomes for postoperative ileus after small intestinal fistula excision in patients with diffuse extensive abdominal adhesions. Front Surg..

[9] Singer M, Deutschman CS, Seymour CW (2016). The third international consensus definitions for sepsis and septic shock (Sepsis-3). JAMA.

[10] Veličković J, Palibrk I, Miličić B (2019). The association of early postoperative lactate levels with morbidity after elective major abdominal surgery. Bosn J Basic Med Sci..

[11] Kraut JA, Madias NE (2014). Lactic acidosis. N Engl J Med.

[12] Boelens PG, Heesakkers FF, Luyer MD (2014). Reduction of postoperative ileus by early enteral nutrition in patients undergoing major rectal surgery: prospective, randomized, controlled trial. Ann Surg.

[13] Lugo G, Arizpe D, Domínguez G, Ramírez M, Tamariz O (1993). Relationship between oxygen consumption and oxygen delivery during anesthesia in high-risk surgical patients. Crit Care Med.

[14] Lemke M, Karanicolas PJ, Habashi R (2017). Elevated lactate is independently associated with adverse outcomes following hepatectomy. World J Surg.

[15] Shimazaki J, Motohashi G, Nishida K, Ubukata H, Tabuchi T (2014). Postoperative arterial blood lactate level as a mortality marker in patients with colorectal perforation. Int J Colorectal Dis.

[16] Li S, Peng K, Liu F, Yu Y, Xu T, Zhang Y (2013). Changes in blood lactate levels after major elective abdominal surgery and the association with outcomes: a prospective observational study. J Surg Res.

[17] Baigrie RJ, Lamont PM, Kwiatkowski D, Dallman MJ, Morris PJ (1992). Systemic cytokine response after major surgery. Br J Surg.

[18] Helander EM, Webb MP, Menard B (2019). Metabolic and the surgical stress response considerations to improve postoperative recovery. Curr Pain Headache Rep..

[19] Wu L, Xiong X, Wu X (2020). Targeting oxidative stress and inflammation to prevent ischemia-reperfusion injury. Front Mol Neurosci..

[20] Younis I (2020). Role of oxygen in wound healing. J Wound Care.

[21] Rodriguez PG, Felix FN, Woodley DT, Shim EK (2008). The role of oxygen in wound healing: a review of the literature. Dermatol Surg.

[22] Guo S, Dipietro LA (2010). Factors affecting wound healing. J Dent Res.

